# Affimers and nanobodies as molecular probes and their applications in imaging

**DOI:** 10.1242/jcs.259168

**Published:** 2022-07-18

**Authors:** Paul Cordell, Glenn Carrington, Alistair Curd, Francine Parker, Darren Tomlinson, Michelle Peckham

**Affiliations:** Astbury Centre for Structural Molecular Biology and the School of Molecular and Cellular Biology, Faculty of Biological Sciences, University of Leeds, Leeds, LS2 9JT, UK

**Keywords:** Affimer, Nanobody, Imaging, Super-resolution microscopy, Phage display

## Abstract

Antibodies are the most widely used, traditional tool for labelling molecules in cells. In the past five to ten years, many new labelling tools have been developed with significant advantages over the traditional antibody. Here, we focus on nanobodies and the non-antibody binding scaffold proteins called Affimers. We explain how they are generated, selected and produced, and we describe how their small size, high binding affinity and specificity provides them with many advantages compared to antibodies. Of particular importance, their small size enables them to better penetrate dense cytoskeletal regions within cells, as well as tissues, providing them with specific advantage for super-resolution imaging, as they place the fluorophore with a few nanometres of the target protein being imaged. We expect these novel tools to be of broad interest to many cell biologists and anticipate them becoming the tools of choice for super-resolution imaging.

## Abstract (precis)

**Summary:** Affimers and nanobodies are small binding proteins that are becoming widely used in cell biology in place of antibodies. We explain how Affimers and nanobodies are generated, used and their advantages for super-resolution imaging.

## Introduction

Antibodies have been widely used by cell biologists to localise proteins within cells using light microscopy since the development of monoclonal technology in the 1970s (Kohler and Milstein, 1975). However, since the development of super-resolution imaging approaches in the past few years, it has become clear that antibodies have several limitations for super-resolution imaging (Carrington et al., 2019; de Beer and Giepmans, 2020; Pleiner et al., 2017; Tiede et al., 2017). Specifically, in immunolabelling applications, their large size (∼150 kDa and ∼15 nm in length for IgG) often hinders effective penetration into tissues and densely packed subcellular structures (Maidorn et al., 2016). Moreover, the large size and flexibility of antibodies places the fluorophore at some randomised or unknown distance from the target proteins, limiting accuracy and precision in super-resolution imaging techniques (Früh et al., 2021).

**Fig. 1.**
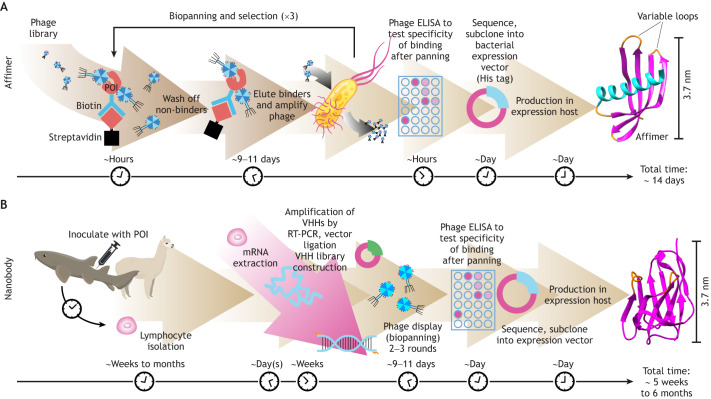
**Generation of Affimers and nanobodies.** (A) Affimers are isolated by screening a phagemid library with a biotinylated protein of interest (POI) on a surface via streptavidin beads. For screening, the biotinylated target protein is bound to plastic-adsorbed streptavidin (first panning round), magnetic bead-conjugated streptavidin (second panning round) and plastic-adsorbed neutravidin (third panning round) (Tang et al., 2017; Tiede et al., 2014). In each panning round, after phage has bound to the immobilised target, the binding matrix is extensively washed, then bound phage is eluted. To amplify binders for subsequent panning rounds, eluted phage is used to infect ER2738 *E. coli* cells, which are then co-infected with M13K07 helper phage to provide the additional proteins needed for phage replication (Vieira and Messing, 1987). The use of different biotin-binding matrices in the screening process acts to reduce carry-through of phage by non-specific interactions. To further enhance specificity, for example to create isoform-specific binders, rounds of negative selection can be added to the procedure as required (Tang et al., 2017). The phagemid DNA of high-affinity clones is sequenced to identify unique binders, which are then subcloned into a bacterial expression vector for Affimer production. (B) Nanobodies from camelids (or vNARs from nurse sharks) are most commonly generated by immunising camelids with the POI, followed by lymphocyte isolation and mRNA extraction (Liu et al., 2018). The VHH-encoding regions are amplified by RT-PCR, and the VHH-encoding sequences are then subcloned into a bacterial expression vector to generate a VHH library. The library is then screened, antigenic binders tested by Phage ELISA and binders sequenced. Unique binders are subcloned into a bacterial expression vector for nanobody production. The approximate timelines are shown underneath.

Several smaller probes have now been developed to overcome the problems of using large antibodies in imaging applications, including Affimers and nanobodies. Affimer reagents are small non-antibody binding proteins, with a molecular mass (12 kDa) ∼10 times smaller than antibodies (Tiede et al., 2017, 2014), and they are less than 4 nm in length (Fig. 1A). Nanobodies are similarly sized (12–14 kDa and less than 4 nm in length) antigen-binding proteins derived from the variable antigen-binding domains of heavy chain only antibodies, which are common in a few animal species, such as camelids and sharks (where they are termed variable new antigen receptors; vNARs) (Dooley and Flajnik, 2006; Dooley et al., 2003; Feige et al., 2014; Feng et al., 2019; Greenberg et al., 1993; Hamers-Casterman et al., 1993; Pleiner et al., 2015). Affimers and nanobodies are a little smaller than GFP (4.7 nm long, 27 kDa), SNAP (∼3.7 nm long, 20 kDa) and Halo (∼5 nm long, 33 kDa) tags. Affimers and nanobodies can thus improve one or more of the potential limitations of antibodies. Their small size enables better penetration of tissues and dense cytoskeletal structures, and places fluorophores much closer to the target protein being imaged in super-resolution microscopy, thereby improving resolution (Tiede et al., 2017).

In this Review, we focus on the properties of Affimers, comparing them to nanobodies in terms of their isolation, applications and their specific advantages, such as small size, stability and high specificity, for super-resolution imaging. We anticipate that the use of these small probes is likely to increase exponentially in the future, and that they will become the standard tools for super-resolution imaging.

## Affimer and nanobody screening and applications

Affimer reagents comprise either a human based-stefin A scaffold (type I) (Stadler et al., 2011) or a plant scaffold (type II). The type II Affimer, originally called Adhirons, comprise a consensus plant phytocystatin protein sequence, genetically engineered to generate a small, monomeric and highly soluble scaffold protein with high thermal stability that lacks disulphide bonds and glycosylation sites (Tiede et al., 2014). Two regions with variable sequence, typically nine residues in length, with randomised amino acid sequences in each, have been inserted into the scaffold to provide the binding interface (Fig. 1). Cysteine residues are omitted from the variable loop sequences, ensuring no cysteines are present in Affimers. This Affimer has been crystallised (PDB ID 4N6U; Tiede et al., 2014) and shown to form the expected cystatin fold (Fig. 1A).

To date, Affimer reagents have been isolated against a wide range of targets, with a variety of binding affinities ranging from the picomolar to micromolar range (Table S1). Affimers that specifically recognise a protein or protein domain of interest are raised by employing an *in vitro* phage display approach (Fig. 1A). The phage display (M13 filamentous phage) library is diverse, comprising over 10 billion unique clones (Tiede et al., 2014). Key for a successful outcome is to use biotinylated protein with high purity and stability at concentrations of ∼4 µM (Tang et al., 2017). Biotinylation is required to attach to the streptavidin-coated substrates for screening. This can be achieved using a BAP(Avi) tag (GLNDIFEAQKIEWHE) inserted onto the N- or C-terminus of the expressed protein (or protein domain) of interest, to enable site-specific and directional biotinylation *in vivo* during expression in *Escherichia coli* (Cull and Schatz, 2000). Alternatively, proteins can be biotinylated *in vitro* once purified. However, in this case biotinylation is random, sequence dependent and can occasionally lead to loss of protein activity or affect structure.

The screening procedure itself is fast (complete within 2 weeks; see Fig. 1A). At the end of the selection procedure, clonal phage is produced and tested by enzyme-linked immunosorbent assay (ELISA) against the biotinylated target to confirm affinity and specificity. The phagemid DNA of clones that bind to the target protein with high affinity is sequenced to identify binders that contain unique variable region sequences. A comprehensive determination of unique binder sequences can be obtained by deep sequencing the pooled phagemid DNA obtained from panning rounds. Affinities are typically in the nanomolar range (Table S1).

The cDNA sequences of Affimer binders (typically we test around ten unique binders) are then subcloned into a bacterial expression vector for Affimer production, introducing a His tag for purification, and any other tags or modifications as required. The Affimers can then be easily expressed and purified from the cytoplasm of *E. coli* for use as affinity reagents (Tang et al., 2017) and are routinely produced in milligram quantities from small (50 ml) cultures of *E. coli*. The advantage of the high thermal stability of Affimers means that bacterial proteins are mostly removed from the bacterial supernatant using a simple heat denaturation (50°C) and precipitation step, and the Affimer can then be further purified using the C-terminal His tag (Tiede et al., 2014). Affimers have been developed at Leeds. The BioScreening Technology Group (University of Leeds, UK) can screen the phage display library to derive Affimers of interest in collaboration with researchers, which can preclude release of sequence information. Affimer libraries utilising a human stefin A as a scaffold have been created by Avacta PLC and can be screened on a commercial basis (https://avacta.com/).

Nanobodies are derived from the variable chain heavy (VHH) domain of antibodies found in Camelidae (camels, llama and alpacas; Muyldermans, 2021b), or variable immunoglobulin new antigen receptor (vNAR) of nurse sharks or closely related species (Dooley et al., 2003; reviewed in Cheloha et al., 2020; Liu et al., 2018). They are most commonly obtained through the immunisation of animals (Fig. 1B), and as a result, can take up to four months to isolate. Nanobodies can also be derived from naïve phage display libraries, but these still require the use of animals for initial generation (Cheloha et al., 2020; Liu et al., 2018). In this respect, the *in vitro* screening for Affimers has the advantage that it does not require the use of animals, and isolation of Affimers is much faster. Moreover, proteins or other antigens that could be toxic to animals can be used in *in vitro* Affimer screens.

More recently, synthetically generated nanobody libraries (which utilise a nanobody-based scaffold with synthetically generated diversity within the binding regions) have been developed (Cabanillas-Bernal et al., 2019; Moutel et al., 2016; Zimmermann et al., 2018; reviewed in Liu and Yang, 2022). Engineering of the nanobody scaffold in synthetic libraries can be exploited to improve biochemical stability and ease of protein folding (e.g. by use of consensus sequences; McMahon et al., 2018; Uchanski et al., 2019). Although screening of synthetic libraries can be faster, some degree of *in vitro* affinity maturation is often be required to obtain high affinity binders (reviewed in Valdes-Tresanco et al., 2022).

Like Affimers, nanobodies are also commonly produced in *E. coli.* Some retain their binding affinity when expressed in the cytoplasm (de Marco, 2020; Olichon and Surrey, 2007; Wagner and Rothbauer, 2020), but others require the formation of a single intra-chain disulphide bond for correct function (de Marco, 2020; Wagner and Rothbauer, 2020). To facilitate this, they are usually expressed in the periplasm, at the cost of a reduced protein yield and limitations on compatible fusion proteins (de Marco, 2020; Pleiner et al., 2015). Affinities of nanobodies for their protein targets are in the picomolar to nanomolar range (Table S2).

Affimers have been used successfully in a wide range of applications, including diagnostics, protein–protein inhibition, and modulation and imaging (Table S1), similar to the more widely used nanobodies (reviewed in Muyldermans, 2021a) (Table S2). For example, Affimers that recognise specific protein conformers (GDP- and GTP-bound states of KRAS) and lock them into distinct conformational states have been isolated (Haza et al., 2021). Similarly, nanobodies that recognise different protein conformers have been isolated. These include the nanobody Nb80, which specifically recognises the ligand-induced active state of the β2 adrenoreceptor (Rasmussen et al., 2011), a nanobody that recognises the GTP-bound form of RHO (Keller et al., 2019), as well as nanobodies that lock proteins into distinct conformational states (reviewed in Uchanski et al., 2020). Moreover, the use of Affimers and nanobodies in imaging applications confer similar advantages, as discussed below.

## Fluorescence imaging applications

To use Affimers in the imaging of fixed cells, we employ a unique cysteine introduced into the N- or C-terminus of the Affimer sequence. The Affimers can then be labelled using maleimide bioconjugation chemistry to attach a single fluorescent dye or biotin. In the latter case, the Affimers are visualised using fluorescent streptavidin (Lopata et al., 2018; Tiede et al., 2017). Although we could also use amine labelling (which labels the ε-amino group of lysine residues), we tend to avoid this method, as lysine residues can be present in one or other of the variable sequences and could interfere with binding of the Affimer with the protein of interest. Affimers have also been generated with SNAP or Halo tags (Keppler et al., 2003; Los et al., 2008), and fluorescent protein or other tags. The cysteine residue has also been used to attach a DNA strand for DNA-points accumulation in nanoscale topography (DNA-PAINT), a form of single-molecule super-resolution imaging (Jungmann et al., 2010; Schlichthaerle et al., 2018). Labelled Affimers are highly stable and can be stored in the fridge for extended periods.

In one of the many examples of Affimers used in imaging (Table S1), we successfully made and characterised four Affimers to filamentous actin (F-actin). Interestingly, only one of these (Affimer 14) labels F-actin in cultured cells fixed using paraformaldehyde. All four Affimers label F-actin in cultured cells fixed using methanol, even though one of these (Affimer 2) bound to actin very weakly (*K*_d_ >10 µM) compared to the remainder (*K*_d_ ∼0.3 µM) as measured by co-sedimentation assays (Lopata et al., 2018). The lower *K*_d_ measured for three out of the four actin Affimers is similar to that reported for fluorescent phalloidin (0.27 µM; Wulf et al., 1979), which is commonly used to label F-actin in cells. Phalloidin does not label F-actin in methanol-fixed cells, making the actin Affimers a useful small-molecule alternative for this application (Lopata et al., 2018). The ability of all four actin Affimers to stain F-actin in cells shows that a weaker binding affinity does not necessarily indicate that an Affimer will not work in an application. Other factors, such as epitope availability in fixed cells, are also likely to be important.

Actin Affimers have also been successfully used to image proteins in live cells, using constructs in which eGFP is linked to the Affimer via a flexible linker and the fusion construct is expressed in a standard mammalian expression vector (Lopata et al., 2018). One actin Affimer (Affimer 6) has since been used in fluorescence polarisation microscopy to measure the orientation of the target protein, as well as its localisation (Sugizaki et al., 2021). To constrain the orientation of the fluorophore dipole, important for fluorescence polarisation, the flexible linker was replaced with a rigid α-helical linker (EAAAK) between the N-terminal helix of the Affimer and the C-terminal 3_10_ helix of superfolder GFP. This construct was crystallised (PDB 7C03), and its binding affinity was measured as ∼300 nM (*K*_d_) with 1:1 stoichiometry for actin, and a strong preference for F-actin (Sugizaki et al., 2021), in agreement with our earlier study (Lopata et al., 2018). This construct was successfully used in fluorescence polarisation microscopy in living cells and starfish oocytes to report on the orientation of F-actin filaments (Sugizaki et al., 2021). In principle, any Affimer that labels proteins in cells could be used in this type of application. Other functional tags, such as the SNAP or Halo tags could also be used, to provide a much wider range of control and diversity in terms of the fluorophore (Haza et al., 2021).

More generally, Affimers can be used in imaging in the same way as has been reported for nanobodies, both using fixed and live cells (recently reviewed in de Beer and Giepmans, 2020). Chromobodies, the nanobody equivalent of fluorescent-protein-tagged Affimers, have been used in live-cell imaging, initially using the GFP nanobody (Rothbauer et al., 2006). A nanobody raised to image actin in plants (Rocchetti et al., 2014) has been developed further to target the nanobody to specific organelles within the cell to image suborganellar actin dynamics (Schiavon et al., 2020). Nanobodies that recognise a peptide tag (15 residues long) on proteins of interest (Traenkle et al., 2020) and nanobodies that replace secondary antibodies (Pleiner et al., 2017), as well as a raft of other useful nanobodies in imaging have also been developed (Traenkle et al., 2020).

In using Affimers for live-cell imaging of target proteins, their expression in live cells needs to be carefully considered, in the same way as nanobodies (chromobodies) or any other live-cell imaging probes (Pleiner et al., 2017). If possible, probes that bind to a functionally important region of its target protein should be avoided if the goal is to image proteins in cells without affecting their function. The effects of expressing any new probe on the cell morphology and protein dynamics need to be carefully monitored. Lifeact, a small peptide fused to eGFP commonly used to image actin in live cells, was originally not thought to interfere with actin dynamics (Riedl et al., 2008). However, it binds to a hydrophobic pocket on F-actin to which myosin and cofilin also bind, explaining some effects on actin and cell morphology seen subsequently (Belyy et al., 2020). Choosing a promoter that drives lower expression levels might be helpful in avoiding or reducing such effects. Over-expression of non-interfering probes that only weakly bind to the target protein might avoid such artefacts, but could lead to a high concentration of unbound probes, decreasing the signal-to-noise ratio. Finally, developing strategies that can deliver probes directly into the cytoplasm of cells without transfection, such as fusion of the constructs to cell-penetrating peptides (recently reviewed in Liu et al., 2022) would also be beneficial, especially for cells that are difficult to transfect.

## Super-resolution imaging

In recent years, fluorescence imaging techniques have advanced from the limits of widefield and confocal microscopy (∼250 nm resolution) to a range of super-resolution microscopy methods with localisation precision down to 1 nm (Schermelleh et al., 2019; Sigal et al., 2018; Valli et al., 2021). As the localisation precision for the positions of fluorescent markers (dye molecules and fluorescent protein tags) has improved, the size of the probe (e.g. the combination of primary and secondary IgG antibodies) has become a limiting factor (Carrington et al., 2019; Früh et al., 2021; Ries et al., 2012).

There are many problems in using the traditional antibody approach. The fluorophore that is imaged is attached to an antibody, which is a long distance away from the target (∼10–30 nm linkage error). Moreover, the fluorophore can adopt multiple different orientations resulting from antibody flexibility. Together, this results in localisation inaccuracy and imprecision (Früh et al., 2021). The labelling ratio (fluorophores per target) is also important for the highest precision approaches, including optimisation of the localisation technique during data acquisition and protein counting during data analysis (Carrington et al., 2019; Früh et al., 2021). A single fluorophore per cellular target is often ideal, and new conjugation techniques for antibody labelling allow this (Früh et al., 2021). Finally, the size of IgG molecule can limit access to epitopes in a crowded environment.

Nanobodies have already been shown to significantly improve super-resolution imaging by reducing the linkage error (the distance between the epitope and fluorophore) and through improved penetration. Nanobodies used in super-resolution imaging include targets, such as fluorescent proteins (Platonova et al., 2015; Ries et al., 2012), short peptide tags, such as BC2 (PDRKAAVSHWQQ) and ALFA (SRLEEELRRRLTE; a short stable α-helix) (de Beer and Giepmans, 2020; Ganji et al., 2021; Götzke et al., 2019; Virant et al., 2018). Nanobodies can also replace anti-IgG secondary antibodies in labelling schemes, including specific labelling of different primary antibodies and in super-resolution imaging (Pleiner et al., 2017; Sograte-Idrissi et al., 2020). Recently, conjugation of a photo-stabilising compound, as well as a dye molecule, has further enhanced the potential of nanobodies in super-resolution imaging where photobleaching or stochastic off-switching of a dye can reduce performance (Schneider et al., 2021). Linkage error in super-resolution images can also be minimized by using unnatural amino acids (followed by labelling with a fluorophore; Arsic et al., 2022), small peptide tags (such as SNAP or Halo; Erdmann et al., 2019) or by direct fusion to fluorescent proteins. However, these methods either require genetic modifications to the samples being imaged or overexpression of tagged proteins.

Like nanobodies, the small size of Affimers makes them ideal for super-resolution imaging, such as stimulated emission depletion (STED) (Willig et al., 2006) and 3D direct stochastic optical reconstruction microscopy (dSTORM) (Heilemann et al., 2009; Rust et al., 2006), and DNA-PAINT (Jungmann et al., 2014). The actin Affimer 14 has been used successfully in both 3D dSTORM and DNA-PAINT (Schlichthaerle et al., 2018). F-actin is ∼8 nm in width. Using DNA-PAINT, the apparent width (from estimating the position of the fluorophore) for F-actin labelled using the actin Affimer (∼3–4 nm in size) was ∼18 nm. In comparison, phalloidin (∼1 nm in size), which binds to the interface between three actin subunits within the actin filament (Mentes et al., 2018; Pospich et al., 2020), gave an apparent width of 13 nm (Schlichthaerle et al., 2018). Part of this increased width, for both the Affimer and phalloidin, is attributable to the DNA strand and the dye. Assuming the Affimer binds to the outside of the actin filament, this demonstrates that Affimer places the fluorophore very close to the target, increasing localisation accuracy.

**Fig. 2.**
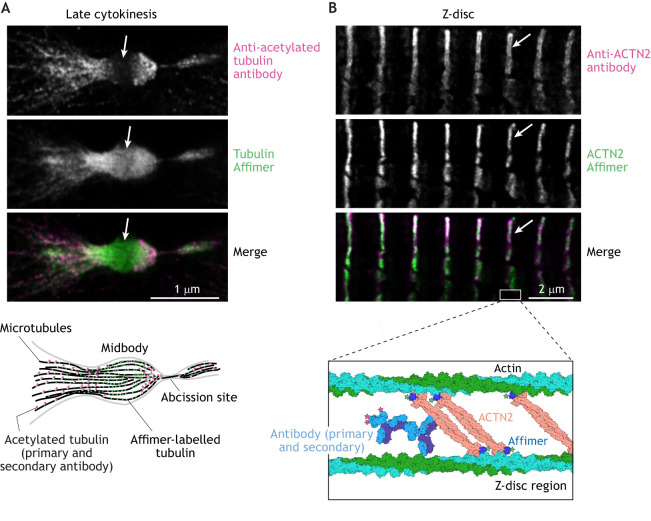
**Advantages of using small probes in imaging.** (A) 2D STED image of a late cytokinetic furrow in cells labelled using an antibody to acetylated tubulin (T7451, Sigma-Aldrich, shown in magenta; signal is from a combination of primary and secondary antibodies) and the tubulin Affimer (Affimer 32, shown in green). These images, summarised in the diagram below, show how densely packed microtubules (25 nm wide) in the central region of the midbody (so-called ‘cut-zone’, arrowed in the STED images) are labelled by the smaller Affimer probe (less than 4 nm long), but not by the more commonly used combination of primary and secondary antibodies (each ∼15 nm long) (see also Tiede et al., 2017). (B) 2D STED image of Z-discs in cardiac muscle tissue, labelled with an antibody to the Z-disc protein ACTN2 (A7732, Sigma-Aldrich; ACTN2) using a combination of primary and secondary antibodies (magenta), and an Affimer to ACTN2 (green). The diagram below illustrates the structures of F-actin, full-length ACTN2 (PDB 4D1E), the ACTN2 Affimer bound to the calponin homology (CH) domains of ACTN2 (PDB 6SWT) (Curd et al., 2021) and primary and secondary antibodies (PDB 1IGY) drawn to scale, for part of the Z-disc. The anti-parallel ACTN2 dimer crosslinks actin filaments (derived from sarcomeres on either side). The combination of primary and secondary antibodies (>20 nm in length) is less able to penetrate the full width of the Z-disc (see arrows on STED images) as illustrated here, accounting for reduced ACTN2 staining in the central region of the Z-disc. The Affimer is much smaller and able to penetrate the entire Z-disc. The restricted localisation of antibodies is noticeable in STED, with a resolution of ∼50 nm, but would not be seen in confocal microscopy, with a resolution of ∼250 nm.

An Affimer to tubulin (Affimer 32) has also been used successfully in super-resolution imaging (Tiede et al., 2017) as have nanobodies to tubulin (Mikhaylova et al., 2015). The apparent width of microtubules labelled with Affimer 32 was ∼47 nm (Tiede et al., 2017), similar to that measured using nanobodies (∼40 nm; Mikhaylova et al., 2015) and much lower than that measured using a directly labelled primary antibody (∼73 nm; Tiede et al., 2017). Affimer 32 labels tubulin in both interphase and mitotic cells (Tiede et al., 2017). A second Affimer (Affimer 7, M.P. and D.C.T.,unpublished) isolated at the same time as Affimer 32 only labels tubulin in mitotic cells. Pulldown experiments using purified tubulin or cell extracts followed by mass spectrometry confirmed that both these Affimers bind tubulin (M.P. and D.C.T., unpublished), but it remains unclear why Affimer 7 only labels mitotic spindles. However, the brain tubulin used in the screen contains multiple isoforms of tubulin and many different post-translational modifications (Roll-Mecak, 2020), raising the possibility that Affimer 7 may be bind to a specific type of tubulin only found in mitotic spindles, and that an Affimer screen could be used to isolate Affimers that recognise specific tubulin isoforms or post-translational modifications. Tubulin Affimer 32 is generally a useful reagent for labelling tubulin in fluorescence microscopy, which works well in standard widefield fluorescence and confocal microscopy, as well as in super-resolution microscopy [STED and dSTORM (Tiede et al., 2017)]. In particular, its small size enables it to label dense microtubule structures, such as the cytokinetic furrow, from which standard antibodies are excluded (Fig. 2A). The small size of nanobodies would also confer such an advantage.

We have further exploited the ability of Affimers to penetrate dense cytoskeletal structures to interrogate the organisation of α-actinin-2 (ACTN2) within the striated muscle Z-disc using 3D dSTORM (Curd et al., 2021). Z-discs are narrow structures (∼80–140 nm from one side of the Z-disc to the other) found at either end of the muscle sarcomere. They are too narrow to be able to use standard fluorescence microscopy to determine how proteins within the Z-disc are organised, as 80–140 nm is below the resolution limit of the light microscope. The organisation of ACTN2 in Z-discs has been characterised by electron microscopy and can either be highly regular in some muscles with a characteristic spacing of ∼19 nm (Burgoyne et al., 2019; Goldstein et al., 1990) or more irregular in others, as recently shown by electron cryo-tomography (Wang et al., 2021). Using a combination of primary and secondary antibodies to image Z-disc proteins is not suitable, as they fail to fully penetrate the Z-disc, whereas an Affimer to ACTN2 does (Fig. 2B). We have also used the same ACTN2 Affimer in 3D dSTORM followed by downstream analysis to reveal a characteristic spacing for ACTN2 of 18.5 nm within the Z-disc, which is close to that observed by electron microscopy (Curd et al., 2021). The ability of the small Affimers to penetrate the Z-disc, combined with the placing of dye molecules close to their target proteins shows the potential of this approach to uncover molecular organisation in dense cytoskeletal structures, such as the Z-disc in cells using dSTORM. Nanobodies to ACTN2 would be expected to work similarly.

Overall, Affimers can make a significant improvement to super-resolution imaging by reducing linkage error to 1–4 nm, controlling the labelling ratio at one dye molecule per probe and binding to targets inaccessible to IgGs (Carrington et al., 2019; Curd et al., 2021; Schlichthaerle et al., 2018; Tiede et al., 2017). They are thus a strong alternative to nanobodies in this type of imaging. Future strategies for super-resolution approaches could improve linkage error, imaging speed and potentially ease of use of protein-based probes. For example, transiently binding coiled coils could be used in place of complementary oligonucleotides, as used in DNA-PAINT as recently described for peptide-PAINT (Eklund et al., 2020). Variations of approaches such as Exchange-PAINT (Jungmann et al., 2014) and madSTORM (Yi et al., 2016) could be developed for Affimers, to enable the controllable dissociation of Affimers from a target (for example mediated by shifts in pH or ionic strength). This would allow washout of Affimers after imaging, prior to introduction of fresh Affimers for a new target, and hence enable optimal multi-target imaging using a single fluorophore species.

## Concluding remarks

Affimers and nanobodies are both examples of small probes that are becoming increasingly widely used in imaging. A specific advantage is their use in super-resolution imaging, which overcomes many challenges associated with traditional approaches, including allowing better penetration into samples and reduced linkage error, which has been well demonstrated for both nanobodies and Affimers. Although each of these small probes has their advantages and disadvantages, Affimers have the specific benefits of employing a completely *in vitro* screen – ease of production and purification from *E. coli*, excellent stability and straightforward labelling for downstream applications. The ability of both nanobodies and Affimers to recognise protein conformers and specific isoforms is likely to lead to new insights into cell biology, as well as highlight their therapeutic potential, and we expect these probes to become more widely used in the future.

## Supplementary Material

Supplementary information
