# Supplementary material for: Affimers and nanobodies as molecular probes and their applications in imaging
Source: J Cell Sci. 2022 Jul 18;135(14):jcs259168. doi: 10.1242/jcs.259168 (PMC9450889; doi:10.1242/jcs.259168)
Supplement: Supplementary information [file joces-135-259168-s1.pdf]

Table S1. Selection of published Affimers, demonstrating range of applications and targets.

Affimer target	Main use	Structures	Imaging	Kd*	Reference
Herceptin	Diagnostics	-	-	12 – 27 nM	(Adamson et al., 2019a)
Therapeutic antibodies	Diagnostics	-	-	-	(Adamson et al., 2019b)
Myoglobin & GFAP glial fibrillary acidic protein	Diagnostics	-	-	pM to nM	(Al-Enezi et al., 2021)
CEA (carcinoembryonic antigen	Diagnostics	-	IF	6 – 34 nM	(Shamsuddin et al., 2021)
Cow Pea Mosaic Virus	Diagnostics	6QOZ	-		(Hesketh et al., 2019)
Clumping factor A (ClfA) virulence factor	Diagnostics		IF	62 nM	(Caudwell et al., 2022)
Glypican-3	Diagnostics	-	-	-	(Xie et al., 2017)
Crimean-Congo hemorrhagic fever orthonairovirus nucleoprotein	Diagnostics, Inhibitor	6ZO0	-	5.7 nM	(Alvarez-Rodriguez et al., 2020)
p300	Protein-protein interaction	5A0O	-	89 - 140 nM	(Kyle et al., 2015)
EPAC1 (Exchange protein directly activated by cAMP 1)	Isoform selectivity	-	IF	-	(Buist et al., 2021)
KRas (GDP and GTP analogue forms)	Isoform selectivity, Inhibitor	7NY8,6YXW,6YR8	-		(Haza et al., 2021)
BCL-2 family proteins	Isoform selectivity	6ST2,6HJL,6STJ	-	90 – 3400 nM	(Miles et al., 2021)
FcγRIIIa	Inhibitor	5ML9, 5MN2	-	217 nM -2.6 µM	(Robinson et al., 2018)
SUMO-1, SUMO-2/3	Isoform selectivity, Inhibitor	5ELJ,5EQL,5ELU	(in cell expression)	35 – 414 nM, and >10 µM	(Hughes et al., 2017)
K6- and K33-linked diUbiquitin	High affinity interactors	5OHM, 5OHV, 5OHL	IF	~20 pM- 1.6 nM	(Michel et al., 2017)
Cholera Toxin B	Protein delivery	-	IF	97-1130 nM	(Balmforth et al., 2021)
ACTN2 (α-actinin-2)	Imaging	6WST	IF, super-res	-	(Curd et al., 2021)
Actin Affimer and Polaris probe (from Actin Affimer)	Imaging	7C03	IF, super-res, live cell imaging, FP	300 nM to >10 µM	(Lopata et al., 2018) (Sugizaki et al., 2021)
Fibrinogen	Modulation	-	(in vitro)	38 – 52 nM	(Kearney et al., 2019)
Transient Receptor Potential Vanilloid 1 (TRPV1) (peptide screen)	Modulation	-	IF	-	(Tiede et al., 2017)
Src2 homology (SH2) domains (p85α, Grb2,7,10,14)	Isoform selectivity	-	-	-	
Tenascin C	In vivo imaging/Diagnostics	-	IHC, in vivo	5.7 nM	
Herpes virus of turkeys (HVT UL49)	Diagnostics	-	IF	1.5 – 7.5 nM	
Tubulin	Imaging (super-resolution)	-	IF, super-resolution	-	
Human epidermal growth factor receptor 4 (HER4)	Imaging (super-resolution)	-	IF, super-resolution	-	
2,4,6- trinitrotoluene (TNT)	Small organic compound detection	-	-	-	

*Kd typically measured by SPR or ITC (except for the Actin Affimer – measured by so-sedimentation with F-actin). IF, immunofluorescence; IHC, immunohistochemistry; FP, fluorescence polarisation. PDB entries for co-crystals (or CryoEM) structures, where available are included.

Journal of Cell Science • Supplementary information

Table S2. Selection of published nanobodies, demonstrating range of applications and targets

Nanobody target	Main use	Structures	Fluorescence Imaging	Kd*	Reference
C4b (complement) (Immunisation)	Inhibitor	5JTW	-	3.2 nM - 14 pM	(De la O Becerra KI, 2022)
Na_v 1.4 and Na_v 1.5 ion channels (Immunisation)	Isoform capture from lysates	7R63	-	40 nM - 60 nM	(Srinivasan et al., 2022)
RHOA NaLi-H1	RhoA-GTP biosensor	-	chromobody	28 nM – 68 nM	(Keller et al., 2019)
BC2-tag (β-catenin) (Immunisation)	Imaging	-	3D dSTORM, chromobody	1.9 nM - ~3.1 nM	(Virant et al., 2018) (Traenkle et al., 2015)
ALFA-tag (Immunisation)	Imaging	6I2G	STED, DNA Paint, IF, chromobody	0.026 nM - 11 nM	(Götzke et al., 2019)
EGFR (Immunisation)	Therapeutics	4KRN, 7OM5, 7OM4	In vivo imaging	<60 nM, 166–276 nM	Reviewed in (Sharifi et al., 2021)
α-synuclein Immunisation)	Diagnostic	-	IHC	1.9 µM - 240 nM (fibril affinity higher)	(Hmila et al., 2022)
HER2 (Immunisation)	Imaging/Diagnostic	-	IF	1.9 nM – 14 nM	(Kijanka et al., 2013)
Actin (Immunisation & NaLi-H1)	Imaging	-	IF, dSTORM (binds G-actin)	-	(Rocchetti et al., 2014) (Moutel et al., 2016)
GFP (Immunisation & NaLi-H1)	Imaging	3OGO, 3K1K	Chromobody, dSTORM, IF	0.23 nM	(Kubala et al., 2010) (Kirchhofer et al., 2010) (Rothbauer et al., 2006) (Moutel et al., 2016)
Tubulin (Immunisation, & NaLi-H1)	Imaging	-	dSTORM, IF	-	(Kesarwani et al., 2020) (Mikhaylova et al., 2015) (Moutel et al., 2016)
Nup proteins (immunisation)	Imaging	6X07 and others	dSTORM, IF, chromobodies	14 pM – 230 nM	(Pleiner et al., 2015) (Nordeen et al., 2020)
β2 adrenoceptor	Structure determination	3POG and others	Fluorescent intrabody Localization Microscopy	1 nM – 100 nM	(Rasmussen et al., 2011) (Gormal et al., 2020)

*Kd typically measured by SPR or ITC. IF, immunofluorescence; IHC, immunohistochemistry. Example PDB entries for co-crystals (or CryoEM) structures, where available are included. NaLi-H1: humanized synthetic single domain antibody library. Remaining nanobodies obtained after initial immunisation.

Journal of Cell Science • Supplementary information

Journal of Cell Science • Supplementary information

References

Adamson, H., Ajayi, M. O., Campbell, E., Brachi, E., Tiede, C., Tang, A. A., Adams, T. L., Ford, R., Davidson, A., Johnson, M. et al. (2019a). Affimer-Enzyme-Inhibitor Switch Sensor for Rapid Wash-free Assays of Multimeric Proteins. *ACS Sens* **4**, 3014-3022.

Adamson, H., Nicholl, A., Tiede, C., Tang, A. A., Davidson, A., Curd, H., Wignall, A., Ford, R., Nuttall, J., McPherson, M. J. et al. (2019b). Affimers as anti-idiotypic affinity reagents for pharmacokinetic analysis of biotherapeutics. *Biotechniques* **67**, 261-269.

Al-Enezi, E., Vakurov, A., Eades, A., Ding, M., Jose, G., Saha, S. and Millner, P. (2021). Affimer-Based Europium Chelates Allow Sensitive Optical Biosensing in a Range of Human Disease Biomarkers. *Sensors (Basel)* **21**.

Alvarez-Rodriguez, B., Tiede, C., Hoste, A. C. R., Surtees, R. A., Trinh, C. H., Slack, G. S., Chamberlain, J., Hewson, R., Fresco, A., Sastre, P. et al. (2020). Characterization and applications of a Crimean-Congo hemorrhagic fever virus nucleoprotein-specific Affimer: Inhibitory effects in viral replication and development of colorimetric diagnostic tests. *PLoS Negl Trop Dis* **14**, e0008364.

Balmforth, M. R., Haigh, J., Kumar, V., Dai, W., Tiede, C., Tomlinson, D. C., Deuchars, J., Webb, M. E. and Turnbull, W. B. (2021). Piggybacking on the Cholera Toxin: Identification of a CTB-Binding Protein as an Approach for Targeted Delivery of Proteins to Motor Neurons. *Bioconjug Chem* **32**, 2205-2212.

Buist, H. K., Luchowska-Stanska, U., van Basten, B., Valli, J., Smith, B. O., Baillie, G. S., Rickman, C., Ricketts, B., Davidson, A., Hannam, R. et al. (2021). Identification and Characterization of an Affimer Affinity Reagent for the Detection of the cAMP Sensor, EPAC1. *Cells* **10**.

Caudwell, J. A., Tinkler, J. M., Johnson, B. R. G., McDowall, K. J., Alsulaimani, F., Tiede, C., Tomlinson, D. C., Freear, S., Turnbull, W. B., Evans, S. D. et al. (2022). Protein-conjugated microbubbles for the selective targeting of S. aureus biofilms. *Biofilm* **4**, 100074.

Curd, A. P., Leng, J., Hughes, R. E., Cleasby, A. J., Rogers, B., Trinh, C. H., Baird, M. A., Takagi, Y., Tiede, C., Sieben, C. et al. (2021). Nanoscale Pattern Extraction from Relative Positions of Sparse 3D Localizations. *Nano Letters* **21**, 1213-1220.

De la O Becerra KI, O. W., van den Bos RM, Xenaki KT, Lorent JH, Ruyken M, Schouten A, Rooijakkers SHM, van Bergen En Henegouwen PMP, Gros P. (2022). Multifaceted Activities of Seven Nanobodies against Complement C4b. *J Immunol* **15**, ji2100647.

Gormal, R. S., Padmanabhan, P., Kasula, R., Bademosi, A. T., Coakley, S., Giacomotto, J., Blum, A., Joensuu, M., Wallis, T. P., Lo, H. P. et al. (2020). Modular transient nanoclustering of activated beta2-adrenergic receptors revealed by single-molecule tracking of conformation-specific nanobodies. *Proc Natl Acad Sci U S A* **117**, 30476-30487.

Götzke, H., Kilisch, M., Martínez-Carranza, M., Sograte-Idrissi, S., Rajavel, A., Schlichthaerle, T., Engels, N., Jungmann, R., Stenmark, P., Opazo, F. et al. (2019). The ALFA-tag is a highly versatile tool for nanobody-based bioscience applications. *Nature Communications* **10**, 4403.

Haza, K. Z., Martin, H. L., Rao, A., Turner, A. L., Saunders, S. E., Petersen, B., Tiede, C., Tipping, K., Tang, A. A., Ajayi, M. et al. (2021). RAS-inhibiting biologics identify and probe druggable pockets including an SII-alpha3 allosteric site. *Nat Commun* **12**, 4045.

Hesketh, E. L., Tiede, C., Adamson, H., Adams, T. L., Byrne, M. J., Meshcheriakova, Y., Kruse, I., McPherson, M. J., Lomonossoff, G. P., Tomlinson, D. C. et al. (2019). Affimer reagents as tools in diagnosing plant virus diseases. *Sci Rep* **9**, 7524.

Hmila, I., Vaikath, N. N., Majbour, N. K., Erskine, D., Sudhakaran, I. P., Gupta, V., Ghanem, S. S., Islam, Z., Emara, M. M., Abdesselem, H. B. et al. (2022). Novel engineered nanobodies specific for N-terminal region of alpha-synuclein recognize Lewy-body pathology and inhibit in-vitro seeded aggregation and toxicity. *FEBS J*.

Hughes, D. J., Tiede, C., Penswick, N., Tang, A. A., Trinh, C. H., Mandal, U., Zajac, K. Z., Gaule, T., Howell, G., Edwards, T. A. et al. (2017). Generation of specific inhibitors of SUMO-1- and SUMO-2/3-mediated protein-protein interactions using Affimer (Adhiron) technology. *Sci Signal* **10**.

Kearney, K. J., Pechlivani, N., King, R., Tiede, C., Phoenix, F., Cheah, R., Macrae, F. L., Simmons, K. J., Manfield, I. W., Smith, K. A. et al. (2019). Affimer proteins as a tool to modulate fibrinolysis, stabilize the blood clot, and reduce bleeding complications. *Blood* **133**, 1233-1244.

Keller, L., Bery, N., Tardy, C., Ligat, L., Favre, G., Rabbitts, T. H. and Olichon, A. (2019). Selection and Characterization of a Nanobody Biosensor of GTP-Bound RHO Activities. *Antibodies (Basel)* **8**.

Kesarwani, S., Lama, P., Chandra, A., Reddy, P. P., Jijumon, A. S., Bodakuntla, S., Rao, B. M., Janke, C., Das, R. and Sirajuddin, M. (2020). Genetically encoded live-cell sensor for tyrosinated microtubules. *J Cell Biol* **219**.

Kijanka, M., Warnders, F. J., El Khattabi, M., Lub-de Hooge, M., van Dam, G. M., Ntziachristos, V., de Vries, L., Oliveira, S. and van Bergen En Henegouwen, P. M. (2013). Rapid optical imaging of human breast tumour xenografts using anti-HER2 VHHs site-directly conjugated to IRDye 800CW for image-guided surgery. *Eur J Nucl Med Mol Imaging* **40**, 1718-29.

Kirchhofer, A., Helma, J., Schmidthals, K., Frauer, C., Cui, S., Karcher, A., Pellis, M., Muyldermans, S., Casas-Delucchi, C. S., Cardoso, M. C. et al. (2010). Modulation of protein properties in living cells using nanobodies. *Nat Struct Mol Biol* **17**, 133-8.

Kubala, M. H., Kovtun, O., Alexandrov, K. and Collins, B. M. (2010). Structural and thermodynamic analysis of the GFP:GFP-nanobody complex. *Protein Sci* **19**, 2389-401.

Kyle, H. F., Wickson, K. F., Stott, J., Burslem, G. M., Breeze, A. L., Tiede, C., Tomlinson, D. C., Warriner, S. L., Nelson, A., Wilson, A. J. et al. (2015). Exploration of the HIF-1alpha/p300 interface using peptide and Adhiron phage display technologies. *Mol Biosyst* **11**, 2738-49.

Lopata, A., Hughes, R., Tiede, C., Heissler, S. M., Sellers, J. R., Knight, P. J., Tomlinson, D. and Peckham, M. (2018). Affimer proteins for F-actin: novel affinity reagents that label F-actin in live and fixed cells. *Sci Rep* **8**, 6572.

Michel, M. A., Swatek, K. N., Hospenthal, M. K. and Komander, D. (2017). Ubiquitin Linkage-Specific Affimers Reveal Insights into K6-Linked Ubiquitin Signaling. *Mol Cell* **68**, 233-246 e5.

Mikhaylova, M., Cloin, B. M., Finan, K., van den Berg, R., Teeuw, J., Kijanka, M. M., Sokolowski, M., Katrukha, E. A., Maidorn, M., Opazo, F. et al. (2015). Resolving bundled microtubules using anti-tubulin nanobodies. *Nat Commun* **6**, 7933.

Miles, J. A., Hobor, F., Trinh, C. H., Taylor, J., Tiede, C., Rowell, P. R., Jackson, B. R., Nadat, F. A., Ramsahye, P., Kyle, H. F. et al. (2021). Selective Affimers Recognise the BCL-2 Family Proteins BCL-xL and MCL-1 through Noncanonical Structural Motifs*. *Chembiochem* **22**, 232-240.

Moutel, S., Bery, N., Bernard, V., Keller, L., Lemesre, E., de Marco, A., Ligat, L., Rain, J. C., Favre, G., Olichon, A. et al. (2016). NaLi-H1: A universal synthetic library of humanized nanobodies providing highly functional antibodies and intrabodies. *Elife* **5**.

Nordeen, S. A., Andersen, K. R., Knockenhauer, K. E., Ingram, J. R., Ploegh, H. L. and Schwartz, T. U. (2020). A nanobody suite for yeast scaffold nucleoporins provides details of the nuclear pore complex structure. *Nat Commun* **11**, 6179.

Pleiner, T., Bates, M., Trakhanov, S., Lee, C. T., Schliep, J. E., Chug, H., Bohning, M., Stark, H., Urlaub, H. and Gorlich, D. (2015). Nanobodies: site-specific labeling for super-resolution imaging, rapid epitope-mapping and native protein complex isolation. *Elife* **4**, e11349.

Rasmussen, S. G., Choi, H. J., Fung, J. J., Pardon, E., Casarosa, P., Chae, P. S., Devree, B. T., Rosenbaum, D. M., Thian, F. S., Kobilka, T. S. et al. (2011). Structure of a nanobody-stabilized active state of the beta(2) adrenoceptor. *Nature* **469**, 175-80.

Robinson, J. I., Baxter, E. W., Owen, R. L., Thomsen, M., Tomlinson, D. C., Waterhouse, M. P., Win, S. J., Nettleship, J. E., Tiede, C., Foster, R. J. et al. (2018). Affimer proteins inhibit immune complex binding to FcgammaRIIIa with high specificity through competitive and allosteric modes of action. *Proc Natl Acad Sci U S A* **115**, E72-E81.

Rocchetti, A., Hawes, C. and Kriechbaumer, V. (2014). Fluorescent labelling of the actin cytoskeleton in plants using a cameloid antibody. *Plant Methods* **10**, 12.

Rothbauer, U., Zolghadr, K., Tillib, S., Nowak, D., Schermelleh, L., Gahl, A., Backmann, N., Conrath, K., Muyldermans, S., Cardoso, M. C. et al. (2006). Targeting and tracing antigens in live cells with fluorescent nanobodies. *Nat Methods* **3**, 887-9.

Shamsuddin, S. H., Jayne, D. G., Tomlinson, D. C., McPherson, M. J. and Millner, P. A. (2021). Selection and characterisation of Affimers specific for CEA recognition. *Sci Rep* **11**, 744.

Sharifi, J., Khirehgesh, M Safari, F. and Akbari, B. (2021). EGFR and anti-EGFR nanobodies: review and update. *J Drug Target* **29**, 387-402.

Srinivasan, L., Alzogaray, V., Selvakumar, D., Nathan, S., Yoder, J Wright, K. M., Klinke, S., Nwafor, J. N., Labanda, M. S., Goldbaum A. et al. (2022). Development of high-affinity nanobodies specific for NaV1.4 and NaV1.5 voltage-gated sodium channel isoforms. *J Biol Chem* **298**, 101763.

Sugizaki, A., Sato, K., Chiba, K., Saito, K., Kawagishi, M., Tomabechi, Y., Mehta, S. B., Ishii, H., Sakai, N., Shirouzu, M. et al. (2021). POLArIS, a versatile probe for molecular orientation, revealed actin filaments associated with microtubule asters in early embryos. *Proc Natl Acad Sci U S A* **118**.

Tiede, C., Bedford, R., Heseltine, S. J., Smith, G., Wijetunga, I., Ross, R., AlQallaf, D., Roberts, A. P., Balls, A., Curd, A. et al. (2017). Affimer proteins are versatile and renewable affinity reagents. *Elife* **6**.

Traenkle, B., Emele, F., Anton, R., Poetz, O., Haeussler, R. S., Maier, J., Kaiser, P. D., Scholz, A. M., Nueske, S., Buchfellner, A. et al. (2015). Monitoring interactions and dynamics of endogenous beta-catenin with intracellular nanobodies in living cells. *Mol Cell Proteomics* **14**, 707-23.

Virant, D., Traenkle, B., Maier, J., Kaiser, P. D., Bodenhöfer, M., Schmees, C., Vojnovic, I., Pisak-Lukáts, B., Endesfelder, U. and Rothbauer, U. (2018). A peptide tag-specific nanobody enables high-quality labeling for dSTORM imaging. *Nature Communications* **9**, 930.

Xie, C., Tiede, C., Zhang, X., Wang, C., Li, Z., Xu, X., McPherson, M. J., Tomlinson, D. C. and Xu, W. (2017). Development of an Affimer-antibody combined immunological diagnosis kit for glypican-3. *Sci Rep* **7**, 9608.

Journal of Cell Science • Supplementary information
